# A Bayesian Change point model for differential gene expression patterns of the DosR regulon of Mycobacterium tuberculosis

**DOI:** 10.1186/1471-2164-9-87

**Published:** 2008-02-22

**Authors:** Yi Zhang, Kim A Hatch, Lorenz Wernisch, Joanna Bacon

**Affiliations:** 1 School of Crystallography, Birkbeck College, University of London, Malet Street, London, WC1E 7HX, UK; 2 TB research, Health Protection Agency, CEPR, Porton Down, Salisbury, SP4 0JG, UK; 3 MRC Biostatistics Unit, University Forvie Site, Robinson Way, Cambridge, CB2 0SR, UK

## Abstract

**Background:**

Low oxygen availability has been shown previously to stimulate *M. tuberculosis *to establish non-replicative persistence *in vitro*. The two component sensor/regulator *dosRS *is a major mediator in the transcriptional response of *M. tuberculosis *to hypoxia and controls a regulon of approximately 50 genes that are induced under this condition.

The aim of this study was to determine whether the induction of the entire DosR regulon is triggered as a synchronous event or if induction can unfold as a cascade of events as the differential expression of subsets of genes is stimulated by different oxygen availabilities.

**Results:**

A novel aspect of our work is the use of chemostat cultures of *M. tuberculosis *which allowed us to control environmental conditions very tightly. We exposed *M. tuberculosis *to a sudden drop in oxygen availability in chemostat culture and studied the transcriptional response of the organism during the transition from a high oxygen level (10% dissolved oxygen tension or DOT) to a low oxygen level (0.2% DOT) using DNA microarrays. We developed a Bayesian change point analysis method that enabled us to detect subtle shifts in the timing of gene induction. It results in probabilities of a change in gene expression at certain time points. A computational analysis of potential binding sites upstream of the DosR-controlled genes shows how the transcriptional responses of these genes are influenced by the affinity of these binding sites to DosR. Our study also indicates that a subgroup of DosR-controlled genes is regulated indirectly.

**Conclusion:**

The majority of the *dosR*-dependent genes were up-regulated at 0.2% DOT, which confirms previous findings that these genes are triggered by hypoxic environments. However, our change point analysis also highlights genes which were up-regulated earlier at levels of about 8% DOT indicating that they respond to small fluctuations in oxygen availability. Our analysis shows that there are pairs of divergent genes where one gene in the pair is up-regulated before the other, presumably for a flexible response to a constantly changing environment in the host.

## Background

*Mycobacterium tuberculosis *is the causative agent for the infectious disease tuberculosis (TB), which kills about 2 million people annually, making it a leading cause of infectious death worldwide. One of the active research areas in the TB field is to investigate the gene regulatory mechanism in *M. tuberculosis *in response to different environmental stimuli it encounters as it adapts and replicates in the human host. However, these adaptations are still poorly understood due to a lack of knowledge of the regulatory cascades controlling the expression of subsets of genes, or regulons. *In vitro *studies aid interpretation of *in vivo *gene expression studies and help to dissect the complex cascades of direct and indirect regulation of regulons.

The success of *M. tuberculosis *as a pathogen is to a large degree due to its ability to persist for long periods within the body, a state referred to as latent or dormant disease. Understanding the environmental cues that initiate latent TB and the subsequent transcriptional response of *M. tuberculosis *will provide markers that are specific to latency, enabling us to refine our search for treatment of this stage of disease. Hypoxia has been identified as a potential stimulus for triggering dormancy. *In vitro *studies have looked at differential gene expression of *M. tuberculosis *in culture under different oxygen tensions and generated lists of genes that are up or down regulated [1-5]. From these studies a two component sensor regulator, known as *dosRS*, was shown to be stimulated by hypoxia. Further mutational studies revealed that 49 genes are controlled by the regulator DosR. Computational sequence analysis revealed a promoter sequence consensus recognised by DosR [3]. DosR-controlled genes may have multiple binding sites.

A study published by Bacon et al. (2004) [1] showed that a low oxygen environment triggers the expression of the DosR regulon in actively dividing cells growing in continuous culture revealing that the DosR regulon may not be specific to latency but will respond to shifts to a low oxygen environment during early infection when *M. tuberculosis *is actively replicating. Different investigations to determine the response of *M. tuberculosis *to low oxygen have observed stimulation of different numbers of DosR-regulated genes and have identified some additional genes putatively controlled by DosR. All these data taken together indicate that the DosR regulon is not a single, synchronous regulon but is in fact differentially regulated depending on the environment. The different numbers of DosR-binding sites upstream of the *dosR*-dependent genes (as many as three) also indicates differential regulation.

To address the question whether DosR-regulated genes are induced all at once or in a temporal cascade, we exposed *M. tuberculosis *to a sudden drop of oxygen from a high level of 10% dissolved oxygen tension (DOT) to a low level of 0.2% DOT and extracted samples from chemostat culture to obtain gene expression values from DNA microarrays. Samples were taken at nine time points over a period of 25 minutes and the corresponding DOT recorded. Through a carefully tailored statistical analysis of the microarray data, we were able to examine the detailed temporal gene expression patterns of the DosR regulon at various oxygen tensions.

Replicates at time points are often not available in sufficient numbers due to the difficulties in obtaining biological samples. Fortunately, in the case of time course data, regression based methods can borrow information for error estimation across time points [6,7]. Here we outline an analysis strategy based on fitting regression splines with step basis functions to time course data. This method allows us to detect at which time point and hence under which oxygen tension each DosR-mediated gene was induced, providing new insights into the genetic program of DosR mediated gene regulation.

For comparison, we also applied the clustering tool STEM [8] which was specifically designed for the analysis of gene expression time course data with few time points. STEM has many statistically attractive features such as significance tests for clusters based on bootstrapping. Nevertheless, this tool failed to detect as many biologically relevant gene inductions as our approach. We chose a Bayesian approach since such an approach provides probabilities that time points are points of induction for particular genes. Probabilities are usually easier to interpret than significance values.

In the following sections we present the results of the change point analysis of the *M. tuberculosis *data and discuss our findings. All methodological aspects, experimental as well as computational, are presented in the methods section, where we also apply our change point model to simulated data to assess its performance.

## Results and Discussion

### Change point analysis of the DosR regulon in the oxygen time-course data

The aim of this study was to determine whether the induction of the DosR regulon unfolds as a cascade of events as the differential expression of subsets of genes is stimulated by different oxygen availabilities rather than a single synchronous event.

To answer this question we analysed the time-course gene expression data of a set of genes/transcription units previously identified as being under the control of DosR. We fitted regression splines with step functions as basis functions to the time-ordered measurements. The positions of the possible steps or change points were placed at time points where measurements were taken. For the statistical analysis we took a Bayesian approach, which allowed us to compare all possible change points in a single probabilistic framework and to obtain probabilities that gene expression is induced at particular time points. Bayesian modelling has two major advantages which were relevant for our aim: 1. it allows us to compare all competing models within a single probabilistic framework; 2. Occam's razor [9] is automatically embodied for finding a trade-off between good fit to the data and model simplicity, that is, fewer change points. The details of the model and priors chosen are outlined in the methods section.

In this study, we focused on 49 genes which were shown previously to be over-expressed under the regulatory control of DosR in a low oxygen environment (0.2% DOT) [3]. These genes are referred to as the DosR regulon in the literature [3,10] and can be further grouped into 37 transcription units (TUs). The transcription unit map of *M. tuberculosis *was obtained from the Biocyc database [11]. The microarray data for the DosR regulon were preprocessed as described in the methods section.

Seven genes of the DosR regulon which showed high experimental variability were filtered prior to analysis according to a criterion detailed in the methods. The change point model was applied to the remaining 42 genes in 32 TUs. In microbial organisms, genes in the same TU are considered co-transcribed, but in practice there might be some discrepancy in expression levels of the genes in the same TU due to the limits in microarray technology and intrinsic biological variation. Typically, expression levels drop off towards the end of an operon due to the reduced efficiency of the transcription process. In the following, we therefore take the first gene in each TU to represent its expression pattern.

#### Change points of expression of transcription units in the DosR regulon

**Figure 1:**
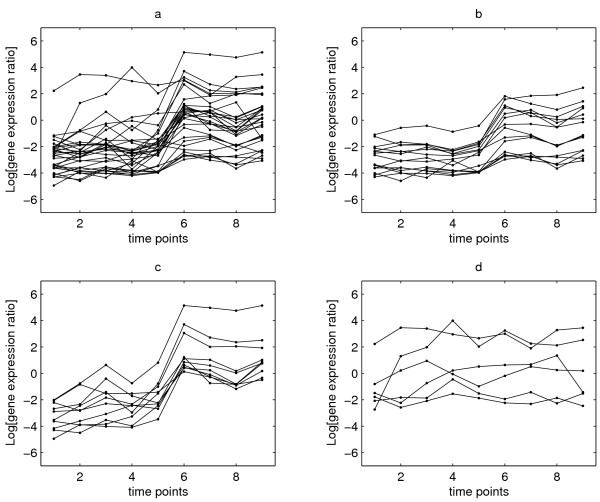
**Temporal patterns of 32 transcription units (TUs) in the DosR regulon**. The temporal patterns of 32 transcription units represented by the first gene in each TU. Subplot a displays the expression profiles of all the 32 TUs. Then we divided these TUs according to whether the detected change points contain time point 6 in each TU as subplots b,c, and d show. (a) All 32 Tus. (b) The TUs with t6 as the only change point. (c) The TUs with more than one change point including t6. (d) The TUs without t6 as the change point

Using the first gene to represent each TU, Figure 1a shows the expression profiles of the 32 TUs. Figures 1b to 1d show the clusters of TUs grouped by the detected change points. Table 1 lists the change points of the first gene in each TU at which the genes showed marked up-regulation except the three time points marked with ↓ where the corresponding genes were down-regulated. Sixteen TUs show marked up-regulation at time point 6 at 0.2% DOT (Figure 1b), another 10 TUs show more complex behaviour than the previous group by including one or two other change points besides the time point 6 (Figure 1c), and the final group (Figure 1d) comprises 6 genes with no change at time point 6, including 4 genes with fairly flat expression levels across all time points.

**Table 1:** The identified change points

ORF	Change-points	STEM No.	Scores.	Motifs
Rv0079	5,6	39	12.4	1
Rv0081	6	39		
Rv0569	6,9	39	9.1	1
Rv0571c	6	N/A	10.3	1
Rv0572c	6	5		
Rv0574c	6	39	13.2	1
Rv1733c	2,6	39	34.8	3
Rv1737c	6	39	46.6	4
Rv1738	2,3	38	46.6	4
Rv1812c	0	36		
Rv1813c	6	39	11.8	1
Rv1997	6	39	17.1	2
Rv2003c	6	39		
Rv2005c	6	39	11.4	1
Rv2006	6	39	11.4	1
Rv2007c	6	39	18.0	2
Rv2031c	2,5,6	39	36.0	3
Rv2032	6	39	36.0	3
Rv2623	6	39	9.42	1
Rv2625c	3,6	39		
Rv2626c	5,6	39	23.2	2
Rv2627c	5,6	39	23.3	2
Rv2628	6,7(↓)	39	18.4	2
Rv2629	6	39	9.97	1
Rv2631	0	N/A		
Rv2830	0	49		
Rv3127	6	5	21.1	2
Rv3129	3,4,9(↓)	14		
Rv3130c	5,6	39	9.7	1
Rv3131	6	39	9.7	1
Rv3134c	3,4(↓),6	39	24.6	2
Rv3841	0	24		

Shown are the change points from the model with the highest probability, STEM cluster number (removed genes indicated by N/A), DosR-binding motif scores and numbers of motifs, and for the 32 TUs. All change points are upregulated except the ones indicated with arrows.

We define change points according to the change point model with the highest probability, including a model with no change points (as calculated by equation 1). An alternative approach to defining change points is to use model averaging and consider all possible change point models weighted by their probabilities. More specifically, the probability that a particular time point is a change point is the sum of the probabilities of all those change point models that contain this time point as a change point. The resulting estimates of probabilities are shown in Table 2. Fortunately, for most genes the two methods of selecting change points agree quite well. The only exception is Rv1738, for which the probability of 0.61 for time point 4 is slightly higher than the probability of 0.51 for time point 3, although the latter and not time point 4 is in the change point model with highest probability. We could have chosen a cutoff on these probabilities to define change points, but it would have necessitated the selection of an arbitrary threshold value, while using the regresion model with the highest posterior probability to determine change points can circumvent the problem.

**Table 2:** Marginal probabilities of change points

ORF	p(t2|D)	(t3|D)	p(t4|D)	P(t5|D)	p(t6|D)	P(t7|D)	p(t8|D)	p(t9|D)
Rv0079	0.27	0.27	0.42	0.92	0.98	0.27	0.31	0.32
Rv0081	0.29	0.41	0.28	0.31	0.73	0.30	0.39	0.37
Rv0569	0.47	0.28	0.25	0.31	0.79	0.30	0.31	0.66
Rv0571c	0.27	0.26	0.26	0.46	0.71	0.29	0.25	0.27
Rv0572c	0.30	0.25	0.27	0.51	0.88	0.35	0.39	0.33
Rv0574c	0.29	0.25	0.25	0.33	0.86	0.30	0.28	0.52
Rv1733c	0.46	0.28	0.28	0.43	1.00	0.29	0.30	0.34
Rv1737c	0.31	0.24	0.25	0.26	0.99	0.30	0.27	0.32
Rv1738	0.98	0.50	0.61	0.43	0.27	0.30	0.25	0.27
Rv1812c	0.30	0.30	0.57	0.48	0.33	0.25	0.31	0.31
Rv1813c	0.29	0.25	0.28	0.28	0.99	0.28	0.33	0.29
Rv1997	0.35	0.29	0.49	0.32	0.75	0.29	0.27	0.32
Rv2005c	0.28	0.25	0.26	0.39	0.93	0.28	0.25	0.30
Rv2006	0.32	0.25	0.26	0.32	0.75	0.31	0.31	0.28
Rv2007c	0.27	0.25	0.26	0.41	1.00	0.39	0.32	0.37
Rv2030c	0.32	0.42	0.34	0.34	1.00	0.29	0.28	0.37
Rv2031c	0.58	0.54	0.32	0.58	0.99	0.26	0.25	0.28
Rv2032	0.27	0.28	0.25	0.38	0.99	0.27	0.26	0.37
Rv2623	0.35	0.28	0.31	0.37	0.99	0.30	0.32	0.50
Rv2625c	0.41	0.51	0.49	0.30	1.00	0.26	0.27	0.31
Rv2626c	0.47	0.39	0.33	0.74	0.99	0.32	0.28	0.27
Rv2627c	0.28	0.41	0.30	0.76	0.96	0.39	0.40	0.31
Rv2628	0.29	0.33	0.27	0.32	0.98	0.77	0.29	0.28
Rv2629	0.36	0.44	0.32	0.28	0.98	0.42	0.29	0.27
Rv2631	0.35	0.30	0.30	0.26	0.29	0.25	0.25	0.33
Rv3127	0.38	0.27	0.28	0.33	0.99	0.38	0.31	0.28
Rv3129	0.32	0.71	0.70	0.39	0.30	0.28	0.33	0.92
Rv3130c	0.28	0.35	0.29	0.69	0.99	0.34	0.27	0.27
Rv3131	0.34	0.33	0.38	0.53	0.91	0.29	0.46	0.33
Rv3134c	0.35	0.63	0.42	0.32	0.88	0.29	0.32	0.48
Rv3841	0.55	0.27	0.31	0.29	0.25	0.40	0.55	0.33

Shown are the marginal posterior probabilites of each time point being a change point for genes in the DosR regulon as derived from model averaging.

In the following, we analyse change points according to whether the binding site pattern for DosR is present in the upstream region of the corresponding gene or not.

#### Change points of transcription units with DosR-binding sites

The details of scores and numbers of DosR-binding sites for each gene were taken from the previous study [3] (see supplementary table 4 in Park et al (2003) [3]). Table 1 displays the number of motifs and scores for each gene. In the case of multiple sites the sum of scores of each site in that TU is listed. In addition to the above previously detected binding sites [3]), We also found several additional highly scoring sites; they are listed in Table 3. The table shows the motif sequences, location and scores of these sites in the promoter region of TUs.

**Table 3:** Additional highly scored DosR-binding motifs found in this study

Sequences	ORFs	Locations	Scores
aTtGGGgtgTAAccTCCacA	Rv1737c	-225	8.5
	Rv1738	-80	8.5
ggCGcGGACaAAtGgCCCgc	Rv2031c	-106	8.3
	Rv2032	-109	8.3
aTtGaGgaccTAagCCCG tt	Rv2623	-128	9.4
gTGGatgacTTTgg tCCCtA	Rv2629	-298	10.0

Lower case characters in sequences show disagreement to the DosR motif consensus TTSGGGACTWWAGTCCCSAA. Locations are the start of the sequences with respect to the translation start site.

**Table 4:** The induced fold changes in the gene expression levels of the two divergently transcribed TUs

ORF	Park et al, (2003) [3]	This study
Rv1737c	8.5	4.9
Rv1738	22.8	16.4
Rv2031c	27.9	20.1
Rv2032	15.1	7.6

The mean induced gene expression fold changes are displayed in Park et al. while in our study the fold changes between t6 and t5 are listed except for Rv1737c whose fold change is between t2 and t1.

**Figure 2:**
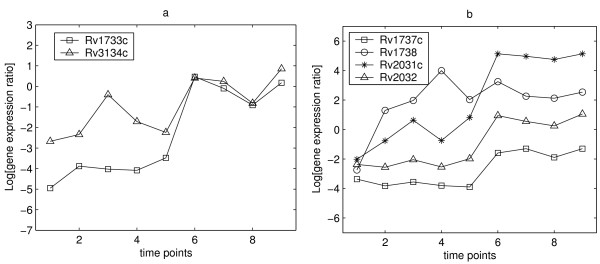
**Upregulation of genes with high binding affinity for DosR**. Figure 2 – displays the expression profiles of four early-upregulated genes and the corresponding divergently transcribed genes sharing common binding motifs. Subplot (a) shows genes with strong binding affinity for DosR up-regulated early (at the time point 2 or 3). Subplot (b) shows two pairs of divergently transcribed genes with strong binding affinity.

There are 23 TUs containing a DosR-binding site. Most of them showed marked up-regulation at time point 6 (at 0.2% DOT) and were up-regulated from time point 5 or 6. There are just four TUs deviating from this pattern in the time-course presented here.: Rv1733c, Rv1738, Rv2031c and Rv3134c, which were up-regulated from time point 2 or 3 on, earlier than the other TUs with DosR-binding sites (see Figures 2a and 2b). Closer inspection of their binding scores shows that they are among the top 6 genes with highest motif scores, suggesting that their early upregulation might be is caused by a strong promoter affinity (see Table 1).

However, among these top 6 genes, there were two TUs, Rv1737c and Rv2032, which showed normal up-regulation from the time point 6, despite their high motif scores, and do not follow the trend of early upregulation of high affinity genes (Figure 2b). An examination of the locations of the motifs of the TUs suggests an explanation for this exception. Gene, Rv1737c shares the motifs with Rv1738, and Rv2031c shares the motifs with Rv2032. It seems that the common motifs shared by two divergently transcribed TUs have more influence on early transcription of only one of the TUs in the pair, while the other is transcribed later with the majority of TUs. This hypothesis is supported by the fold-changes in the gene expression levels of the above two divergently transcribed TU pairs. In both our study and the previous study [3], it is observed that the changes in the induced gene expression levels of Rv1738 and Rv2031c were much stronger than those of Rv1737c and Rv2032 (see Table 4). Mapping the transcriptional start sites would provide useful information about the differential expression of these divergent genes. The evidence of early induction shown in the time-course we present here, combined with the high motif scores and high levels of induction of these two genes provides an interesting hypothesis that warrants further investigation. It is intriguing that Rv1738 and Rv2031c are induced by 8% DOT and their expression level then decreases to increase again when the DOT reaches 0.2%. The expression levels would be expected to continue to increase with DOT decrease. It is possible that these genes are subject to dual regulation. Recently it has been shown that sensor kinases, *dosS *and *dosT*, function in response to subtly different signals [12]. Gene, *dosS *functions as a redox sensor, whereas *dosT *functions as a hypoxia sensor demonstrating the existence of dual control within the DosR-regulon.

#### Change points of transcription units without DosR-binding sites

There are nine TUs in the DosR regulon which contain no obvious DosR-binding sites in their promoter region. Four of these TUs (Rv1812c, Rv3841, Rv2631, Rv2830c) show little changes in their expression profiles and no change points have been assigned to them by our algorithm. These genes were not induced in continuous culture during steady-state growth under low oxygen conditions (0.2% DOT) in our previous study [1]. The explanation for the genes' induction in other studies and the lack of induction in our two low oxygen studies using the chemostat could be that these genes do not respond to low oxygen, but have responded to other environmental factors in previous batch models (such as nutrient-limitation or NO stress) where the environmental conditions are fluctuating. The gene Rv3129, however, shows a gene expression pattern quite distinct from that of other DosR-controlled genes. As the change point analysis reveals, the expression level went up at time points 3 (at 6% DOT) and 4 (at 4% DOT) and then levelled off until the last time point at which it was markedly down-regulated. Four more TUs (Rv0081, Rv0572c, Rv2003c, Rv2625c) lacking DosR-binding sites were up-regulated from time point 6 (at 0.2% DOT). This might be due to indirect DosR regulatory control. The details of detected change points and binding motif scores for each gene are displayed in Table 1.

### Clustering TUs in DosR regulon using STEM

For comparison, we also analysed the expression profiles of the first genes in the 32 TUs using the clustering software STEM, which was designed for clustering short time series gene expression data [8]. Briefly, STEM first selects *m *template profiles from all possible profiles on levels -*c*, ..., 0, ..., *c*, for a constant *c*, that are as different as possible. Each gene expression profile is then assigned to one of the *m *template profiles, namely the one to which its correlation is highest. Once clusters are formed a permutation test provides significance values for clusters of unusual size.

The default settings of 50 for the number of template profiles and 5 for the number of distinct expression levels was used. Two genes, Rv2631 and Rv0571c, were excluded by STEM prior to clustering as the expression changes were smaller than the default threshold of one unit (that is, a two fold change in expression levels) in STEM. In our change point analysis, gene Rv2631 was also flagged as non-differentially expressed, while Rv0571c was identified as having a change point at time point 6. Gene Rv0571c was shown to contain DosR-binding motifs in the promoter region in the previous study [3].

**Figure 3:**
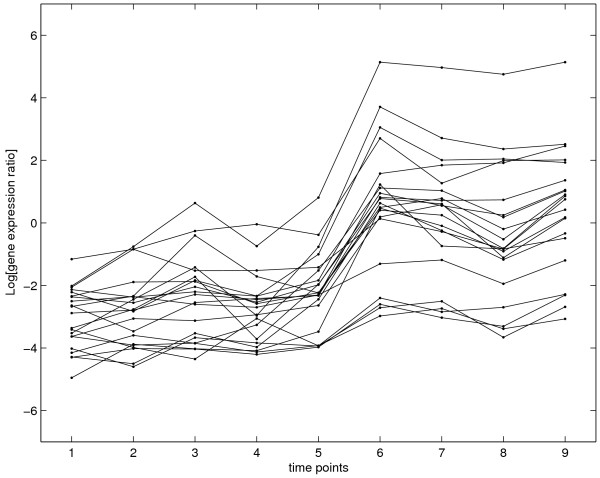
**Expression profiles of TUs in cluster 39 of STEM analysis**. This Figure shows the expression profiles of 23 TUs in the cluster 38 obtained from STEM analysis. It can be seen that TUs with different change points were clustered into the same cluster as described in the text. As in the change point analysis, the expression profile of each TU is represented by its first gene.

STEM analysis results in 7 non-empty clusters. The cluster memberships of genes are listed in Table 1 (NA indicates that the genes have been removed in the filtering step). The largest cluster contains 23 TUs and their expression profiles are shown in Figure 3. The remaining 7 TUs were assigned to 6 clusters of which 5 contain only one gene. Three of these singular genes have no change points according to the Bayesian change point model. However, for genes Rv3127 and Rv0572c our change point analysis showed a clear change point at time point 6. In STEM, the significance was determined by the size of the actual clusters and the expected number of genes in each cluster based on the permutation tests. Hence the clusters with a larger number of genes seem more likely to be significant while small sized profiles are penalised.

Both the Bayesian model and STEM analysis show that the expression levels of 23 genes in cluster 39 (Figure 3) were induced and share a similar profile but the Bayesian model further reveals the subtle differences in the expression patterns of these genes, for instance, despite being in a similar profile, the expressions of some genes (for example, Rv1733c and Rv2031c) started to change as early as time point 2 while others remained unchanged until time point 6.

## Conclusion

Changes in oxygen tension encountered by the pathogen during the time-course experiment are likely to reflect those at the different sites of the host's body. For example, upper lobes of the lung, the most oxygenated region could correspond to the aerobic condition at the first time point. Other oxygen deprived regions, such as the granuloma and the macrophage, are akin to the experimental conditions with low oxygen tensions at subsequent time points. The change point analysis of the oxygen time-course has revealed for the first time that the induction of the majority of dosR-regulated genes is triggered by a very low DOT (0.2%) and not a minor shift in DOT. These data also reveal that these genes respond to a sudden drop in oxygen tension in addition to the adaptive response previously demonstrated.

However, our change point analysis has highlighted genes within the DosR regulon that do not fit the currently recognised profile for this regulon. Some genes in the DosR regulon were already up-regulated at DOT levels as high as 8%. This analysis suggests These data indicate that not only do some of the *dosR*-dependent genes respond to very low levels of oxygen, associated with the hypoxic environment of the granuloma in advanced disease, they also respond to small fluctuations in oxygen availability. Such fluctuations are more likely to be encountered in the early stages of infection when the organisms are actively replicating. Previous chemostat studies have shown that the majority of the DosR regulon is induced under low oxygen in exponentially growing cells. We have also identified genes (for example Rv1812c), the expression of which does not appear to respond to low oxygen at all in either our time-course or steady-state studies and yet has previously been shown to be induced by hypoxic conditions and controlled by DosR [3].

Our analysis has added further information to the previous study [13] indicating that acr-co-regulated genes Rv1737c/Rv1738 and Rv2031c/Rv2032 are temporally regulated. In each pair of divergent genes one of the genes is up-regulated prior to the induction of the other gene in the pair. Static experiments previously have not revealed these subtleties, which may be required for a flexible response to a constantly changing environment in the host. These observations, however, are based on a single time course experiment and further experiments are necessary to confirm our preliminary findings.

The clustering algorithm STEM [8] used here failed to tease out subtly different expression patterns of the TUs in the DosR regulon detected by the change point model. The change point model is proving to be a valuable complementary analysis tool to current clustering methods and is able to provide additional insight into the dynamics of gene expression. The temporal regulatory patterns of the DosR regulon observed *in vitro *has provided some clues as to the spatial regulatory patterns of the DosR regulon *in vivo*. Functions now need to be assigned to these genes to enable us to further understand how these genes are employed during the infection process. This in turn will lead to an understanding of whether the entire regulon serves as a marker of latent disease or whether it is just a proportion of the regulon that is specific to this disease state.

## Methods

### Time-course experiments

*M. tuberculosis *H37Rv was grown in continuous culture to steady state under aerobic conditions (10% DOT) at pH 6.9 and 37°C, in a chemostat, which was controlled by a Brighton Systems controller unit. Cells were grown under carbon-limitation at a dilution rate of 0.03h-1 and a mean generation time of 23 hr. The culture was switched from continuous to batch growth just prior to the start of the time-course.

The set point on the chemostat controller was reduced from 10% DOT to 0.2% DOT and the oxygen level dropped to the lower set point over 15 minutes. The approach of using continuous culture was adopted in order to generate cells that were growing with the same mean generation time under defined and controlled conditions. The controlled chemostat system was also advantageous during the time-course as the oxygen level could be monitored throughout the time-course. The time point at which each sample was taken was not dictated by the time that had lapsed between each time point (although this was also recorded), but the DOT in the culture (see Table 5).

**Table 5:** The percentage of dissolved oxygen tension (% DOT) at each time point

Time points	%DOT
t1(0 minutes)	10
t2(1 minutes)	8
t3(4 minutes)	6
t4(8 minutes)	4
t5(10 minutes)	2
t6(15 minutes)	0.2
t7(17 minutes)	0.2
t8(20 minutes)	0.2
t9(25 minutes)	0.2

Microarray RNA was extracted from cell samples (10 ml) taken at each time point according to the method described previously [1]. Three separate labelling reactions were carried out on each RNA sample, giving three arrays for each time point using the microarray method described previously [1]. In summary, each Cy5-labelled cDNA generated from an RNA sample was co-hybridised with Cy3-labelled DNA generated from *M. tuberculosis *H37Rv genomic DNA.

The resulting gene expression data used in this analysis were log 2 transformed intensity ratios, defined as intensity values of Cy5-labelled cDNA relative to Cy3-labelled DNA. Prior to log 2 transformation, the arrays were preprocessed by the software Bluefuse [14] to estimate signals and subtract background. Then the same normalisation procedure as used in the previous work [15] was applied to the microarray data to reduce experimental noises.

The medians of three array replicates were taken at each time point for each gene. To reduce the potential for false identification of change points caused by experimental errors, we excluded highly-variable genes from the DosR regulon with coefficients of variation (CV) larger than 0.8 which is the 85th percentile of all the CVs, at more than two time points, since high CV at multiple time points indicates poor measurement reproducibility, which might distort the true gene expression profiles. The CVs were computed by dividing the means of gene expression levels (before log transformation) of the three replicates by the corresponding standard deviations at each time point.

The time-course microarray data used in this study were deposited in the Bugs (Accession No:E-BUGs-54) and Array Express databases (Accession No:E-BUGs-54).

### Model specification

In this study, the main goal was to explore whether the DosR-controlled genes are a single synchronised regulon or are in fact induced under dynamic oxygen tensions at different time points. To this end, we applied and adapted a Bayesian change point model with bases in the form of step functions as described in the work [16].

The choice of basis functions depends on the shape of the underlying curves. In the context of microarray data analysis, though the curves under the null hypothesis simply take the form of a constant line, there are many patterns of gene expression changes across the time points. Higher order splines are sometimes used in the analysis of time-course data, but we did not consider them appropriate for a short time-course experiment as they tend to overfit the data [8]. In this analysis, we assumed the underlying time-course gene expression trajectories of *dosR*-dependent genes were in the form of step functions which either are constant lines, i.e., unchanged gene expressions across time points, or which have a few break points where gene expressions are markedly altered. This assumption seems adequate to capture the expression patterns of these genes, according to the previous studies of DosR regulon gene expressions [2,3,17] and the data observed in this experiment.

Let $y_{j}^{i}$ denote the log ratio of gene expression intensities for gene *i *at time point *j*, where *i *= 1, ...., *G *(genes), and *j *= 1, ..., *J *(time points). The gene expression measurements ${\left(y_{j}^{i}\right)}_{j=1}^{J}$ can be modelled as a function of time points ${\left(t_{j}\right)}_{j=1}^{J}$:

$$y_{j}^{i}=g_{i}(t_{j})+\varepsilon_{j}^{i}$$

Where *g*_*i *_is a regression function to be learned from the data and is defined as:

$$g_{i}(t_{j})=\beta_{0}^{i}+\sum_{q=1}^{Q}\beta_{q}^{i}B_{iq}$$

*B *= (*B*_*iq*_) is the *J *× (*Q *+ 1) design matrix specified below and $\beta^{i}=(\beta_{0}^{i},....\beta_{Q}^{i})$ is a vector of regression coefficients. We assume that the error term $\varepsilon_{j}^{i}$ is an independent random variable from a Gaussian distribution with zero mean and variance $\sigma_{i}^{2}$. *Q *is the number of knots or change points. This assumption is routinely made in statistical microarray studies when applied to logarithmic intensity levels. To avoid the situation where the parameters in the model are under-determined, *Q *is constrained to be equal or smaller than *J *- 1. We assume knots *x*_1_, ..., *x*_*Q *_at which the step regression function can change. The design matrix is then

$$B=\left(\begin{array}{cccccc} 1 & f_{1}(t_{1}) & . & . & . & f_{Q}(t_{1})\\ 1 & f_{1}(t_{2}) & . & . & . & f_{Q}(t_{2})\\ . & . & . & & & .\\ . & . & & . & & .\\ . & . & & & . & .\\ 1 & f_{1}(t_{J}) & . & . & . & f_{Q}(t_{J}) \end{array}\right)$$

where *f*_*q*_(*t*_*j*_) is the step basis function defined as:

*f*_*q*_(*t*_*j*_) = *I*(*t*_*j *_≥ *x*_*q*_)

*I*(.) is the indicator function which returns 1 for a true argument and 0 otherwise. We allowed knots *x*_*q *_only at *t*_2_, ..., *t*_*J *_(and hence *Q *= *J *- 1), that is, at any time point except the first (a knot at the first time point would correspond to a constant function which is represented by the overall mean)

**Figure 4:**
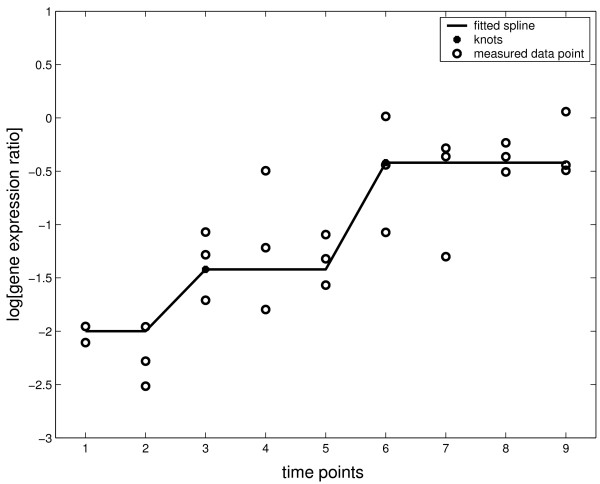
**An example of spline line curve fitting**. In the plot, the replicated measurements (circles) of the time-course were simulated from a step-wise spline function with added independent normal errors. The solid line shows the fits to the data with knots *x*_*q *_at the time points t3 and t6.

The method essentially is to fit an optimal curve to the time-course profiles by determining the locations of the knots where the underlying measurements significantly change. Figure 4 illustrates an example of fitting a step-wise spline curve to the simulated time-course measurements. It can be seen from the plot that the time-course profile changed at the time point 3 and 6. The spline curve fitted the time-course profile well by putting the knots at the corresponding time points.

In this study, we adopted a Bayesian approach to find the optimal curves fitting the data which has three basic steps:

(a) provide priors to all the unknown parameters in the model;

(b) compute the likelihood of the data (marginal data likelihood);

(c) select the best model for optimal curving fitting.

In the above step a, we have an opportunity to incorporate some previous knowledge into the priors of unknown parameters, e.g., error variances.

#### Prior specification

We assumed that all models were equally probable using a uniform prior on the model space:

$$p(M)={\left(\sum_{i=0}^{J-2}\left(\begin{array}{c} J-1\\ i \end{array}\right)\right)}^{-1}={\left(2^{\left(J-1\right)}-1\right)}^{-1}$$

where *i *is the number of change points in the model *M*. If *i *= 0, the model *M *represents a constant fit to the time-course gene expression trajectory, indicating non-differential gene expression. The maximal number of change points allowed in a model *M *is *J *- 2 (to keep the number of parameters below the number of observations). The number of possible locations of change points is *J *- 1 (no change point was allowed at the first time point).

To compute the model likelihood in an analytical form, we used a Gaussian inverse Gamma conjugate prior for ***β ***and *σ*^2^:

*p*(***β ***| *σ*^2^,*υ*) = *N*(**0**, *υσ*^2^*I*_*Q*+1_)

We set *υ*^-1 ^= 0.1. To assess the sensitivity to *υ *we also analysed the data of DosR-controlled genes using *υ*^-1 ^= 0.01 and obtained similar but slightly more conservative results. The prior of ***β ***is conditional on the regression variance *σ*^2^, which has the following prior distribution:

*σ*^-2 ^~ Gamma (*a, b*)

with hyperparameters *a *= 1, and *b *= 0.5. The hyperparameters were chosen based on the estimates of sample variance components of the aforementioned 49 DosR-controlled genes using restricted maximum likelihood (REML) approach implemented in the software YASMA [18]. To estimate the possible range of regression variances, we estimated the sample variance components of the first two samples, and of the first and the last samples in the microarray time-course data respectively. The REML estimates of the sample variance components are 0.026 and 1.28 respectively. This prior distribution also encompasses our weak belief over the sample variance components larger than two as the right tail of the prior distribution *P*(*σ*^2 ^≥ 2) = 0.22 and *P*(0.026 ≤ *σ*^2 ^≤ 1.28) = 0.68.

#### Marginal data likelihood of Bayesian change point model

Choosing conjugate priors enables us to compute the marginal data likelihood analytically [16]:

$$p(D|M)=\frac{\left|V^{*}{|}^{1/2}{\left(b\right)}^{a}\Gamma (a^{*}\right)}{\left|V{|}^{1/2}\pi^{(Q+1)/2}\Gamma (a\right)}{\left(b^{*}\right)}^{-a^{*}}$$

where

*V *= *υI*_*Q*+1_

V* = (*V*^-1 ^+ *B'B*)^-1^

m* = (*V*^-1 ^+ *B'B*)^-1^*B'****y***

a* = *a *+ (*Q *+ 1)/2

b* = *b *+ {***y'y ***- (m*)'(V*)^-1^m*}/2

The marginal data likelihood *p*(*D *| *M*) can be interpreted as the probability that randomly selected parameter values from the model class would generate data set *D*. It is a key quantity in Bayesian model selection as it automatically implements Occam's principle to balance model simplicity and model fit. When using the uniform model prior, a Bayes factor can be computed as the ratio of marginal data likelihoods: *p*(*D *| *M*_*i*_)*/p*(*D *| *M*_0_), in which *M*_*i *_denotes any model with at least one change point while *M*_0 _represents the model under the null hypothesis, i.e., a model fitting a constant line to the gene expression profile. In the following, we select the models with the largest marginal data likelihood (equation 1) to define change points in the time-course. This is equivalent to choosing a model with the highest Bayes factor and also the highest posterior probability of competing models if a uniform model prior is used.

#### Promoter analysis of transcription units in the DosR regulon

The following DosR-binding motif has been identified in the promoters of several genes in the DosR regulon:

5'-TTSGGGACTWWAGTCCCSAA-3'

It has a palindromic structure as expected of the LuxR family of response regulators [3]. Mutation within this binding motif in gene Rv2031c prevents DosR-binding [3]. To further establish the link between the expression patterns of TUs in the DosR regulon and their promoter affinity, we searched sequences (300 bps) upstream of each transcription unit to compute the scores of the DosR-binding motifs using the method described in the study [3].

#### Posterior probability of change points

The Bayesian setting of the model enables us to obtain the posterior probability of a time point being a change point by integrating over all models containing this point in the basis:

$$p(t_{q}|D)=\sum_{i:t_{q}\in M_{i}}p(M_{i}|D)=\sum_{i:t_{q}\in M_{i}}\frac{p(D|M_{i})p(M_{i})}{\sum_{j}p(D|M_{j})p(M_{j})}$$

where *p*(*t*_*q *_| *D*) is the posterior probability of a change point at time point *t*_*q*_, *p*(*M*_*i *_| *D*) is the posterior probability of model *M*_*i *_containing *t*_*q *_in the basis and *j *indexes all the possible models.

### Simulation study

Prior to analysis of the oxygen time-course microarray data, the change point model was tested on the simulated data. In the simulation study, synthetic data were used to compare an approach using Bayesian change point models with a simple fold-change approach. The synthetic data were generated by adding different levels of noise indicated by signal to noise ratio (SNR) to the true mean gene expression level at each time point:

***y***^*i *^= ***μ ***+ **ε**^*i*^

in which ***y***^*i *^is a vector of simulated observed expression levels for gene *i*, ***μ ***is a vector of assumed "true" gene expression levels, which will be specified below, and error terms $\varepsilon_{j}^{i}\sim N(0,\sigma_{\varepsilon }^{2})$, with $\sigma_{\varepsilon }^{2}=\frac{\sigma_{\mu }^{2}}{\text{SNR}}$, where $\sigma_{\mu }^{2}$ is the variance of the ***μ ***vector and SNR a specified signal to noise ratio.

We set up 4 different experiments each with 500 simulated profiles over 9 time points. We tried to make the simulated profiles look similar to log2 transformed gene expression data. To compare different methods, we computed the true positive rate (TP) and the false positive rate (FP) as follows:

$$\begin{array}{c} \text{TP}=\frac{\text{number of detected true change points}}{\text{number of total true change points}}\\ \text{FP}=\frac{\text{number of detected false change points}}{\text{number of total non-change points}} \end{array}$$

For example, in the first simulation study where each gene has one change point, the number of total true change points was 500 = 500 × 1 and of total true negatives 4000 = 500 × (9 - 1).

In the fold-change approach, we selected change points when the fold change between a time point and its previous one exceeded the predetermined threshold. Here we used 2 and 1.5 fold-change as thresholds. In our Bayesian change point model approach, we selected the model with the highest marginal data likelihood using equation 1. Then all the time points used as the knots in the basis of that model were regarded as the change points.

#### Simulation 1

In the first synthetic dataset, each gene had a two-fold change corresponding to the log-ratio changing by unity between the time point 5 and 6. A gene was regarded as differentially expressed if its expression level changed by two-fold or more. The simulated log transformed gene expression profile is:

***μ ***= (-1, -1, -1, -1, -1, 0, 0, 0, 0)

#### Simulation 2

To investigate how robust the methods are to small gene expression variations, we generated synthetic data with fold change of 1.5 and log transformed the true gene expression levels:

***μ ***= (-0.585, -0.585, -0.585, -0.585 -0.585, -0, -0, -0, -0)

#### Simulation 3

Since gene expression levels often vary, we simulated a dataset with two change points of varying fold changes. The log transformed true gene expression profiles are:

***μ ***= (*δ*_1_, *δ*_1_, *δ*_1_, 0, 0, 0, *δ*_2_, *δ*_2_, *δ*_2_)

in which *δ*_1 _and *δ*_2 _are randomly drawn from a uniform distribution in [-0.58, -2] corresponding to fold changes ranging from 1.5 to 4 before log transformation.

#### Simulation 4

As a further point of testing the model, we generated five sets each with hundred genes of constant expression profiles:

***μ ***= (-2, -2, -2, -2, -2, -2, -2, -2, -2, -2)

Different levels of noise were added onto each set with variances *σ*^2 ^= 0.1^2^, 0.2^2^, 0.3^2^, 0.4^2^, 0.5^2^. In this simulation, any genes detected with change points were counted as false positives.

#### Results of simulation

All the above simulations except for simulation 4, contain genes with one or two change points. Tables 6 to 8 display true positive rates and false positive rates resulting from different methods applied to each synthetic dataset respectively. It can be seen that the fold-change approach seems very sensitive to the amount of noise and the changes in expression levels between the time points and also to the chosen arbitrary threshold. When the threshold of 1.5 fold change was lower than the true underlying fold change of 2 and the noise was small relative to the true signal, the fold change approach performed as well as the Bayes model. For example, the performance of fold change of 1.5 is similar to the Bayes model, as Table 7 shows. Nevertheless, with increasing amounts of noise or the true fold change close to the predetermined threshold, the fold-change approach either picked up a high proportion of false positives or failed to detect true positives. The Bayesian change point model appears less affected by the magnitude of true fold changes and more robust to noise.

**Table 6:** True positive and false positive rates for simulation 1

		Δ_*fold *_= log 2	Δ_*fold *_= log 1.5	*p*(*D *| *M*)
SNR = 100	TP	0.54	1.0	1.0
	FP	0	0	0
SNR = 16	TP	0.48	1.0	1.0
	FP	0	0	0
SNR = 4	TP	0.53	0.86	0.92
	FP	0.01	0.1	0.02

The true gene expression change is two-fold. Δ_*fold *_denotes the thresholds used in the fold change approach, and *p*(*D *| *M*) the marginal data likelihood as computed in equation 1.

**Table 7:** True positive and false positives rates for simulation 2

		Δ_*fold *_= log 2	Δ_*fold *_= log 1.5	*p*(*D *| *M*)
SNR = 100	TP	0	0.50	1.0
	FP	0	0	0
SNR = 16	TP	0	0.49	1.0
	FP	0	0	0
SNR = 4	TP	0.03	0.50	0.81
	FP	0	0.01	0.02

The true gene expression change is one-and-a-half folds. Δ_*fold *_and *p*(*D *| *M*) are the same as in Table 6.

**Table 8:** True positive and false positive rates for simulation 3

		Δ_*fold *_= log 2	Δ_*fold *_= log 1.5	*p*(*D *| *M*)
SNR = 100	TP	0.69	0.98	0.97
	FP	0	0	0
SNR = 16	TP	0.68	0.95	0.93
	FP	0	0.02	0
SNR = 4	TP	0.66	0.90	0.82
	FP	0.05	0.2	0.05

The true gene expression change ranges from one-and-a-half to four folds. Δ_*fold *_and *p*(*D *| *M*) are the same as in Table 6.

Table 8 shows the results of simulation 3, a more challenging situation where expression levels varied with mixed fold-changes ranging from as small as less than 1.5 to 4. Furthermore, there were two change points in a non-monotonic trend for each gene in this simulation. When the SNR was reduced to 4 in simulation 3, the fold-change method with 1.5 folds picks 85% true positives equivalent to 894 true change points but the number of false positives also increased dramatically to 695 false change points (20%FP), which is too high for practical purposes while the Bayesian model detected 815 true positives and 182 false positives (5%FP).

**Figure 5:**
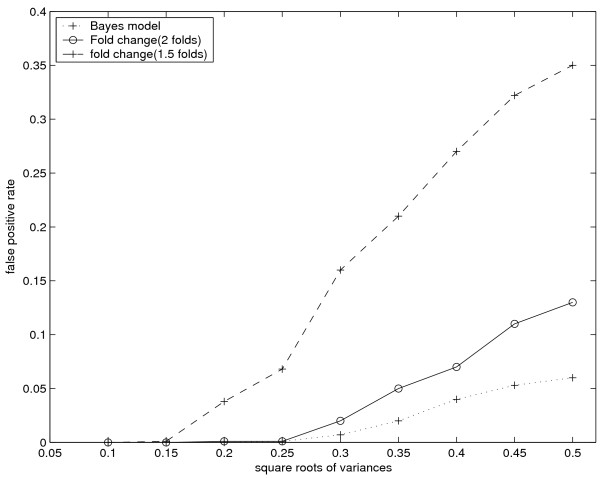
**False positives in simulation 4**. In this simulation no gene has a change point over standard deviation of added noise. The plot shows the proportion of genes falsely identified with change points.

Figure 5 shows the proportion of genes which were falsely identified with change points using different methods in simulation 4 in which only constant gene profiles were generated. As the figure shows, the results of simulation experiment 4 further demonstrate the robustness of the Bayesian model to various levels of noise.

## Authors' contributions

YZ and LW developed the statistical approach and YZ carried out the data analysis. JB and KAH carried out the chemostat culture and microarray experiments. YZ, JB, and LW contributed equally to the manuscript and approved the manuscript.
